# Comparative Chloroplast Genomics of Acanthaceae with a Focus on Medicinal Plant *Thunbergia grandiflora* Roxb.: Unveiling Adaptive Evolution, Diversification Mechanisms and Phylogenetic Relationships

**DOI:** 10.3390/biology15020137

**Published:** 2026-01-13

**Authors:** Yanlin Zhao, Wei Wu, Jinzhi Chen, Qingqing Lin, Chang An, Guoqiang Chen, Yanfang Zheng, Mingqing Huang, Yanxiang Lin

**Affiliations:** 1 College of Pharmacy, Fujian University of Traditional Chinese Medicine, Fuzhou 350122, China; 2230408028@fjtcm.edu.cn (Y.Z.); 2240408016@fjtcm.edu.cn (W.W.); chen2250456012@163.com (J.C.); linqq597@126.com (Q.L.); yfzheng@fjtcm.edu.cn (Y.Z.); hmq1115@126.com (M.H.); 2 Fujian Provincial Key Laboratory of Haixia Applied Plant Systems Biology, College of Life Sciences, Fujian Agriculture and Forestry University, Fuzhou 350002, China; ancher0928@163.com; 3 College of Ocean Food and Biological Engineering, Jiangsu Ocean University, Lianyungang 222005, China; gqchen@jou.edu.cn

**Keywords:** *Thunbergia grandiflora*, Acanthaceae, chloroplast genome, selection pressure analysis, phylogeny

## Abstract

The medicinally and ornamentally valuable genus *Thunbergia* faces taxonomic uncertainty, while certain Acanthaceae species are threatened by habitat loss, underscoring the need for chloroplast genome studies to support conservation efforts. The chloroplast genome of *Thunbergia grandiflora* was sequenced and assembled. Additionally, 28 Acanthaceae species with significant medicinal value were selected for comparative genomic analysis. Based on the chloroplast genome data of Acanthaceae species, this study conducted phylogenetic and comparative evolutionary analyses. The results preliminarily support a systematic framework that divides Acanthaceae into eight tribes within five subfamilies. Concurrently, the study revealed significant inverted repeat (IR) region structural variations. A clear correspondence was observed between the contraction of IR length and the topological structure of the phylogenetic tree. In particular, species within the genus *Strobilanthes* exhibited significant contraction in their IR regions, which corresponded consistently with their tendency to cluster into an independent clade in the phylogenetic tree. This suggests that structural variation in the IR regions may be closely associated with the evolutionary divergence of this group. SSR analysis revealed a prevalent mononucleotide A/T repeat dominant pattern across Acanthaceae species. Furthermore, selection pressure analysis detected positive selection acting on multiple key genes, including *rbcL*, *rps3*, *rps12*, *cemA*, and *ycf4*, suggesting that these genes may play important roles in the adaptive evolution of Acanthaceae. This study reveals that the chloroplast genomes of Acanthaceae exhibit distinctive characteristics in phylogenetic architecture, dynamic variations in IR regions, and adaptive evolution of key genes, providing important molecular insights for understanding the mechanisms underlying species diversity and for the conservation of medicinal resources within this family.

## Simple Summary

Blue Sky Vine *Thunbergia grandiflora* (Acanthaceae) holds medicinal and economic value, yet its genomic background remains poorly understood. Habitat loss and overexploitation further threaten Acanthaceae species, underscoring the need for genomics-based conservation. Its chloroplast was sequenced and comparatively analyzed with those of 27 representative species spanning 24 genera across all five subfamilies of Acanthaceae, and a robust phylogenetic tree encompassing 68 Acanthaceae species was constructed. Key findings revealed distinct genetic variation within the family, including a notable contraction in the inverted repeat region of *Strobilanthes* species, which phylogenetically supports their unique clade formation. The identification of simple sequence repeats, hypervariable regions, and adaptive evolution patterns provides molecular insights into species differentiation and environmental adaptation. By integrating published chloroplast genomes and coding sequences from seven additional genera, this work enhances phylogenetic resolution and establishes a genomic foundation for classification, evolutionary studies, and conservation planning in Acanthaceae. Further integration of nuclear and morphological data will help clarify the diversification mechanisms within this family.

## 1. Introduction

**Figure 1:**
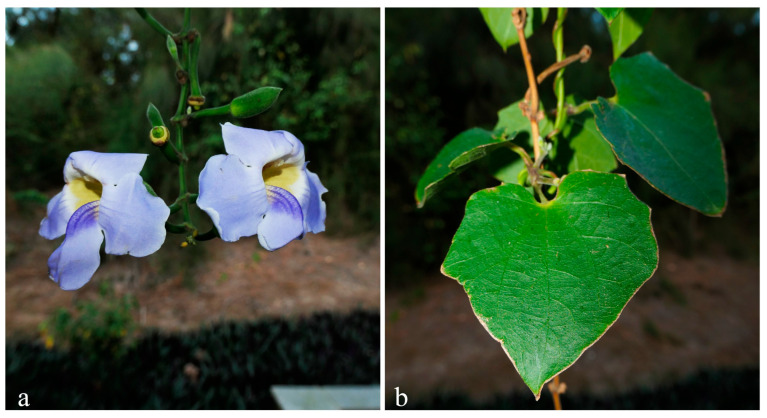
Morphological characteristics of *Thunbergia grandiflora*. (**a**) Bluish-purple flowers; (**b**) Subcordate leaves.

The genus *Thunbergia* Retz. is classified within the subfamily Thunbergioideae of the family Acanthaceae Juss belonging to Lamiales Bromhead [1]. Due to its distinctive morphological characteristics, this subfamily was elevated by some taxonomists to the rank of a separate family, Thunbergiaceae [1,2]. Clarifying the taxonomic status of Thunbergioideae is therefore of significant importance for systematic studies of its constituent species. *Thunbergia* comprises approximately 100–150 species with a distribution ranging from tropical Asia to Australia and sub-Saharan Africa [3]. There are 9 species and 2 subspecies found in southern China [4]. However, plants of the Acanthaceae family are predominantly distributed in tropical and subtropical regions. These areas are characterized by intense human activity and rapid land-use changes, rendering their native habitats highly vulnerable to degradation and loss [5,6]. For instance, the mangrove species *Acanthus ebracteatus* Vahl and some species within the genus *Justicia* L. are now endangered due to habitat constraints, uncontrolled anthropogenic harvesting, and the extensive use of herbicides in rural areas, leading to the near-exhaustion of their wild populations [7,8]. Consequently, there is an urgent need to undertake scientific assessment and conservation efforts based on genomic information [9,10]. Plants of this genus are predominantly climbing herbs or shrubs, characterized by bilabiate corollas. The fruit is a large, woody, rostrate capsule or a globose to ellipsoid drupe with a long apical beak. It distinctly lacks hooked retinacula. The majority of species within the genus, such as *Thunbergia laurifolia* Lindl., *Thunbergia coccinea* Wall., *Thunbergia fragrans* Roxb. and *Thunbergia alata* Bojer ex Sims, exhibit distinctive inflorescence morphology and confer significant ornamental merit [4]. Also known as The Blue Sky Vine, *Thunbergia grandiflora* Roxb. grows into a robust plant with dense foliage and abundant blooms. It provides extensive ground cover, producing long, pendulous racemes and an extended flowering period, all of which contribute to its high overall ornamental value. The leaves are typically ovate, broadly ovate, to cordate in shape and exhibit palmate venation. Flowers are usually solitary in the leaf axils or arranged in racemose inflorescences. The bracts are small and ovate, while the corolla tube is white and the limb bluish-purple (Figure 1). Each flower bears four stamens, with filaments gradually widening toward the base. The fruit is a woody capsule, globose at the base and tapering into a long beak at the apex [4].

Many species within the genus *Thunbergia* possess significant medicinal value. Traditional records indicate that different species or plant parts are used for their properties, such as clearing heat and detoxifying, reducing inflammation and relieving pain, calming the liver and clearing damp-heat, as well as relaxing tendons and promoting blood circulation. They are traditionally applied in the treatment of various conditions including carbuncles, traumatic injuries, headaches, dizziness, damp-heat diarrhea, and snakebite wounds [11,12,13]. Furthermore, in regions such as Yunnan, the flowers of various *Thunbergia* species are consumed by local ethnic communities as nutraceutical ingredients [14]. *Thunbergia* plants contain diverse bioactive constituents, including flavonoids, alkaloids, phenolic acids, coumarins, and iridoids [15,16]. Modern pharmacological studies have confirmed that these compounds possess significant antitumor, anti-inflammatory, antimicrobial, antioxidant, and immunomodulatory properties [17,18,19]. These significant medicinal and economic values underscore the critical importance of sustainable utilization and conservation of their wild resources. Accurate species identification, clarification of phylogenetic relationships, and assessment of genetic diversity within *Thunbergia* are not only fundamental for their development and utilization but also serve as the scientific basis for formulating effective conservation strategies to prevent resource depletion.

The chloroplast genome (cp DNA) is a circular double-stranded DNA molecule (typically 120–160 kb in length) that serves as the genetic material within chloroplasts of photosynthetic organisms, including plants and algae [20]. Compared to the nuclear genome, the chloroplast genome exhibits greater genetic independence and conservation, demonstrating semi-autonomous inheritance [21,22]. Although chloroplast genome encodes genes essential for its own functions, it relies on the nuclear genome for replication and repair processes [23]. Structurally, the chloroplast genome is highly conserved in terms of gene content, organization, and composition, with rare occurrences of rearrangement events [24]. It typically encodes approximately 110–130 genes, while non-coding regions display significant sequence variability [22]. The chloroplast genome of plants, characterized by its conserved and variable regions as well as maternal inheritance, serves as a crucial tool in molecular pharmacognosy research [25]. The non-coding regions of chloroplast genomes exhibit higher variability than coding regions, making them particularly useful for distinguishing closely related species, while their maternal inheritance avoids interference from genetic recombination due to hybridization, thus clearly reflecting species evolutionary history [26,27]. In molecular pharmacognosy, chloroplast genomes are widely applied in species identification and authentication, and kinship analysis, assessment of genetic diversity in medicinal resources, as well as synthetic biology and active compound production [28,29,30]. Research on plant chloroplast genomes serves as the foundation for molecular pharmacognosy studies, providing essential genetic information for molecular identification and germplasm exploration of Chinese herbal medicines. Nowadays there is an urgent need to enrich chloroplast genome datasets of medicinal plants [31].

Although extensive genomic sequencing efforts have been conducted on Acanthaceae species, significantly enriching the genomic database of this family, a comprehensive phylogenetic tree based on complete chloroplast genomes and a systematic comparative analysis of chloroplast genomes across the family remain lacking [32,33,34]. Our previous research has already sequenced and published the cp genome of *Asystasia gangetica* (L.) T. Anderson now this study employed Illumina high-throughput sequencing technology to sequence the chloroplast genomes of *T. grandiflora*. Combined with 66 complete chloroplast genomes covering 24 genera of Acanthaceae obtained from the National Center for Biotechnology Information (NCBI) database, maximum likelihood (ML) phylogenetic trees were constructed using both complete chloroplast genome sequences and coding sequences (CDS). Within the phylogenetic framework, 28 Acanthaceae species of notable medicinal value were selected for comparative genomic analysis. Compared to prior studies, this research newly includes representative species from the genera *Hypoestes* Sol. ex R. Br., *Ecbolium* Kurz, *Pachystachys* Nees, *Asystasia* Blume, *Hygrophila* R.Br., *Chroesthes* Benoist, and *Lepidagathis* Willd., thereby filling gaps in previous phylogenetic analyses and further enriching the phylogenetic framework of Acanthaceae. Moreover, this work establishes a foundational resource for future research in *Thunbergia*, including chloroplast genetic engineering, genetic lineage reconstruction, biodiversity assessment, traditional Chinese medicine germplasm verification, sustainable resource utilization, and selective breeding.

## 2. Materials and Methods

### 2.1. Genomic DNA Extraction and Sequencing

Plant samples of *T. grandiflora* were collected from Xiamen City, Fujian Province, China (24°27′9″ N, 118°5′38″ E). Healthy and pest-free fresh young leaves were selected, flash-frozen in liquid nitrogen, transported to the laboratory, and stored at −80 °C for further use. Voucher specimens were deposited in the Herbarium of Fujian University of Traditional Chinese Medicine. Total genomic DNA was extracted from young leaves of *T. grandiflora* using the DNA Quick Plant System (TIANGEN BIOTECH Co., Ltd., Beijing, China). The concentration and purity of the extracted DNA were assessed by agarose gel electrophoresis and UV spectrophotometry. Upon meeting the criteria for library construction, the DNA was used to prepare a sequencing library. Paired-end sequencing with a read length of 150 bp was performed on the Illumina platform (Novogene Bioinformatics Technology Co., Ltd., Tianjin, China).

### 2.2. Chloroplast Genome Assembly and Annotation

The obtained Illumina data were quality-checked using Fastqc v 0.11.9 [35]. Raw data were processed with Fastp v0.23.4 [36] software to remove low-quality reads, short sequences, and adapter sequences, yielding high-quality clean data. The clean data were assembled using Getorganelle v1.7.7.0 [37] with *Thunbergia erecta* (Benth.) T. Anders (MZ555773) as the reference genome [38]. The assembled chloroplast genome was validated by mapping clean reads back to the assembly using bam v0.7.17 [39] and samtools v1.18 [40]. Genome annotation was performed using CPGAVAS2 http://47.96.249.172:16019/analyzer/annotate (accessed on 5 August 2025) [41] and GeSeq website https://chlorobox.mpimp-golm.mpg.de/index.html (accessed on 5 August 2025) [42], followed by manual curation to remove redundant annotation information. And tRNAscan-SE v2.0 [43] software was utilized to verify the tRNA annotations, leading to the final acquisition of annotated information. The complete chloroplast genome of *T. grandiflora* was mapped using the Chlorobox online tool and subsequently submitted to National Center for Biotechnology Information (NCBI) with its final annotation, where it was assigned a GenBank accession number (PX516301). In this study, functional annotation for the *T. grandiflora* species was performed using the Chloroplast Genome Analysis Suite (CGAS) [44].

### 2.3. Selection of Species for Analysis

Additionally, based on the established phylogenetic framework, we selected the chloroplast genomes of 28 Acanthaceae species with significant medicinal value for comparative analysis. The selected species included: including *Dicliptera montana* Lindau (MK833946) [45], *Peristrophe japonica* (Thunb.) Bremek. (MW411448) [46], *H. ypoestes forskaolii* (Vahl) R.Br. (ON398071) [47], *Justicia procumbens* L. (MN848245) [48], *Justicia patentiflora* Hemsl. (MN848248), *Rungia pectinata* (L.) Nees (MK946456) [49], *Clinacanthus nutans* (Burm. f.) Lindau (MH778102), *Ecbolium viride* (Forssk.) Alston (MW482858) [33], *Pachystachys lutea* Nees (OP546128) [50], *Asystasia gangetica* (PP680763) [51], *Pseuderanthemum haikangense* C. Y. Wu & H. S. Lo (MT747169) [33], *Strobilanthes tonkinensis* Lindau (MW525447) [52], *Strobilanthes cusia* (Nees) Kuntze (MG874806) [53], *Hygrophila ringens* (L.) R. Brown ex Spreng. (OQ871461) [54], *Ruellia elegans* Poir. (OQ564492), *Echinacanthus longzhouensis* H. S. Lo (MH045156), *Barleria lupulina* Lindl. (ON768802) [34], *Chroesthes longifolia* (Wight) B.Hansen (OR555753), *Lepidagathis incurva* Buch-Ham. ex D. Don (PQ576719), *Andrographis paniculata* (Burm. f.) Wall. ex Nees (KF150644) [55], *Acanthus ilicifolius* L. (MW174172) [56], *Blepharis ciliaris* (L.) B.L.Burtt (MK548576) [57], *Aphelandra knappiae* Wassh. (MH909777) [58], *Avicennia marina* (Forssk.) Vierh. (MT012822) [59], *Avicennia officinalis* L. (MW160467), *T. erecta* (MZ555773) [38], *T. grandiflora* (PX516301), *Staurogyne concinnula* (Hance) Kuntze (ON553916) [60].

### 2.4. Comparative Analysis of Chloroplast Genomes

CGAS [44] was used to calculate GC content and the lengths of the four characteristic partitions. MISA v2.1 [61] tool was used to detect simple sequence repeats (SSRs) in the chloroplast genome of 28 Acanthaceae species, with the minimum thresholds for mononucleotide to hexanucleotide re-peats set at 10, 5, 4, 3, 3, 3, respectively. The boundary variations in the quadripartite structure were identified as the primary source of chloroplast genome divergence. Boundary comparisons of large single-copy (LSC), small single-copy (SSC), and Inverted Repeat (IR) regions among 28 Acanthaceae species were conducted using CPJSdraw v1.0.0 [62]. To assess whether the variation in IR region length was associated with phylogenetic relationships among the studied Acanthaceae species, the approach described by Abdullah et al. was followed [63]. Regarding the correlation between IR length variation and phylogeny, the IR length was calculated for each species, and a phylogeny-informed diagram was generated using an R script v4.5.2. IR lengths were mapped onto the phylogenetic tree using a color gradient to visualize patterns across clades. Pairwise absolute differences in IR length and patristic distances derived from the phylogenetic tree were also computed. Finally, a Mantel test with 9999 permutations was performed to evaluate the correlation between IR length differences and phylogenetic distances. Sequence similarity analysis of 28 Acanthaceae species was performed using mVISTA https://genome.lbl.gov/vista/mvista/about.shtml (accessed on 29 September 2025) [64] in Shuffle-LAGAN mode with *D. montana* (MK833946) [46] as the reference genome to detect potential rearrangements and inversions. Nucleotide diversity (π) analysis of shared coding sequences (CDS) and intergenic spacers (IGS) across the complete chloroplast genomes of 68 Acanthaceae species was performed using DnaSP v0.5.10 [65] with a window length of 800 bp and a step size of 200 bp.

### 2.5. Codon Usage Bias Analysis

The relative synonymous codon usage (RSCU) reflects the deviation of codon usage from random expectation, with variation among species linked to gene expression levels Protein-coding genes from 28 Acanthaceae species were extracted using Phylosuit v1.2.2 [66], filtered to retain only CDS ≥ 300 bp with standard start (ATG) and stop (TAA/TAG/TGA) codons. RSCU frequencies were calculated using MEGA7.0 [67], and synonymous codon usage patterns were analyzed through the CodonW [68] online tool, where RSCU values > 1 indicated preferentially used codons, values < 1 represented under-represented codons, and values = 1 suggested no codon usage bias [69].

### 2.6. Analysis of Codon Usage Bias Influencing Factors

The effective number of codons plot (ENC-plot) analysis was used to visually represent codon usage patterns in species genes and evaluate the influence of mutation and natural selection on codon usage bias [70]. The ENC values were calculated using the Chips tool in Emboss v6.6.0. [71]. A two-dimensional scatter plot was constructed with GC3 content (%) as the *x*-axis and the ENC values as the *y*-axis, while the standard curve representing expected ENC values (indicating codon bias influenced solely by mutation) was plotted in the same scatter plot. The parity rule 2 plot (PR2-plot) analysis was employed to examine the equilibrium of third codon position mutations [72]. The third base of codons was identified as the critical factor influencing codon usage bias. A scatter plot was generated with G3/(G3 + C3)|4 as the *x*-axis and A3/(A3 + T3)|4 as the *y*-axis. The neutrality plot analysis was utilized to assess factors influencing codon usage bias, where synonymous mutations predominantly occurred at the third codon position while non-synonymous mutations mainly affected the first or second positions, with non-synonymous mutations exhibiting lower mutation rates [73]. A standard curve was plotted with GC3 content of each gene as the *x*-axis and GC12 (the average of GC1 and GC2) as the *y*-axis.

### 2.7. Selection Pressure Analysis

Selection pressure, as an evolutionary driving force, functioned as an external factor promoting organismal adaptation to environmental conditions [74]. In this study, amino acid sequences were first aligned using MAFFT v7.52 [75], followed by mapping the alignment results to nucleotide sequences using the pal2nal v14 package to obtain codon-aligned CDS sequences. The concatenated gene sequences were then processed with Phylosuite v1.2.2 [66], where 68 sequences were divided into pairwise fa. files and converted to axt format using the parseFastaIntoAXT.pl v1.2 script. Based on the YN model, KaKs_Calculator3.0 [76] was employed to compute synonymous mutation rates (Ks), nonsynonymous mutation rates (Ka), and Ka/Ks ratios. Following biological research conventions, selection pressure types were determined by Ka/Ks ratios: positive selection (Ka/Ks > 1), neutral selection (Ka/Ks = 1), or purifying selection (Ka/Ks < 1). To further investigate selective pressures, HyPhy v2.5.7 was employed [77], applying the Branch-Site Unrestricted Statistical Test for Episodic Diversification (BUSTED) [78] to detect gene-wide signals of episodic positive selection across phylogenetic branches, and the Mixed Effects Model of Evolution (MEME) [79] to identify site-specific episodic diversifying selection affecting particular lineages. Codon alignments for each gene were performed using MUSCLE v5 [80]. For the identified positive selection sites, the secondary structure of proteins containing these sites was predicted using PSIPRED https://bioinf.cs.ucl.ac.uk/psipred/ (accessed on 21 December 2025) [81], and their three-dimensional structural models were constructed via the SWISS-MODEL https://swissmodel.expasy.org/interactive (accessed on 21 December 2025) [82] online server. The maximum likelihood phylogenetic tree reconstructed in this study was incorporated into the analysis.

### 2.8. Phylogenetic Analysis

To elucidate the phylogenetic relationships within Acanthaceae, 67 complete plastid genomes (*Asystasia gangetica* (L.) T. Anderson [51] has been already published in our previous study) spanning 24 genera of this family were obtained from the NCBI database for phylogenetic reconstruction. Four Bignoniaceae species were selected as the outgroup: *Tanaecium tetragonolobum* (Jacq.) L.G.Lohmann (NC_027955) [83], *Dolichandra cynanchoides* Cham. (NC_037460) [84], *Adenocalymma acutissimum* Miers (NC_037455) [84], and *Adenocalymma biternatum* (A.Samp.) L.G.Lohmann (NC_036496) [85]. Chloroplast genome sequences were aligned using MAFFT v7.520 [75], and the alignments were subsequently trimmed with trimAL v1.4 [86] under a heuristic strategy. Following the generation of a high-quality multiple sequence alignment, the optimal nucleotide substitution model (GTR + F + I + G4) was determined with IQ-TREE v2.0.3 [87]. Based on this model, a maximum likelihood (ML) tree was constructed using RAxML v8.2.12 [88] with 1000 bootstrap replicates. To enable comparison with the phylogeny based on complete chloroplast genomes, 52 shared CDS sequences were extracted from 68 Acanthaceae species. These sequences were aligned with MAFFT v7.520 [75] and subjected to codon-aware alignment correction using MACSE v2.07 [80] and Gblocks v0.91 [89]. All CDS were concatenated and partitioned using Phylosuit v1.2.2 [66]. The best substitution model for each gene partition was selected via the ModelFinder module in IQ-TREE v2.0.3 [87], and a final ML tree was inferred with 1000 bootstrap replicates.

### 2.9. Divergence Time Estimation

To reconstruct the evolutionary history of the major lineages within Acanthaceae, this study estimated the divergence times within Acanthaceae using the mcmctree program in the PAML v4.9 software package [90], with a chloroplast genome-based maximum likelihood tree as the input phylogeny. For time calibration, five fossil nodes were constrained. Four species from Bignoniaceae (*D. cyanachoides*, *T. tetragonolobum*, *A. acutissimum*, and *A. biternatum*) were selected as outgroups. Fossil evidence was incorporated by applying a lognormal prior (offset = 45.0, mean = 1.5, standard deviation = 0.5) to the Bignoniaceae node, which was fixed at 49.50 Ma [91]. Key nodes within Acanthaceae were calibrated using fossil data: a constraint of 23.8–5.3 Ma was applied to the tribe Barlerieae; the most recent common ancestor (MRCA) of *Strobilanthes* and *Ruellia* was set to 12.01–10.39 Ma [92]; the crown age of Acanthoideae was calibrated to 27.82 Ma using an *Acanthus* fossil; and the stem age of Avicennioideae was constrained to 37.8 Ma with an *Avicennia* fossil [93]. The analysis was performed under a GTR + G model with an independent rates clock. The mean substitution rate (rgene_gamma) was estimated using the baseml program in PAML v4.9 [90]. The mcmctree analysis was run twice independently, each with 1 × 10^5^ generations, sampling every 100 generations, and the first 4 × 10^4^ generations were discarded as burn-in. Convergence and reliability of the results were assessed using Tracer v1.7 [94], with all parameters exhibiting effective sample sizes (ESS) greater than 200.

## 3. Results

### 3.1. Structural Characteristics of the Thunbergia grandiflora Chloroplast Genome

**Figure 2:**
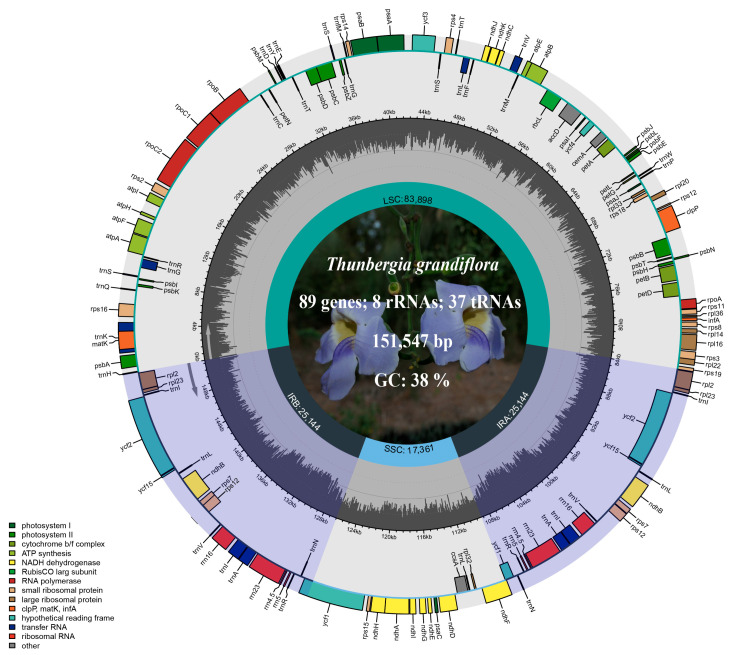
Visualization of *Thunbergia grandiflora* chloroplast genome map with annotations. The outermost circle indicates gene orientation, with genes of different functional categories color-coded as shown in the lower left corner of the circular plot. The innermost circle represents the size and boundaries of the four structural regions of the chloroplast genome.

The complete chloroplast genome of *T. grandiflora* was determined to be 151,547 bp in length, with an overall GC content of 38%, exhibiting a typical quadripartite structure (Figure 2). This structure consisted of a large single-copy (LSC) region of 83,898 bp, a small single-copy (SSC) region of 17,361 bp, and a pair of inverted repeat (IR) regions, each 25,114 bp in length.

A total of 132 genes were annotated in the *T. grandiflora* chloroplast genome, comprising 87 protein-coding genes, 8 rRNA genes, and 37 tRNA genes (Table 1). Functionally, these genes were classified into four categories: photosynthesis, self-replication, other functional, and genes of unknown functions. Among the 132 annotated genes, 15 genes were found to contain one intron, including 9 protein-coding genes (*ndhA*, *ndhB*, *petB*, *petD*, *atpF*, *rpl16*, *rpl2*, *rps16*, *rpoC1*) and 6 tRNA genes (*trnA-UGC*, *trnG-UCC*, *trnI-GAU*, *trnK-UUU*, *trnL-UAA*, *trnV-UAC*). Additionally, 3 genes (*rps12*, *clpP*, *ycf3*) were identified to contain two introns. It is noteworthy that the *rps12* gene is a trans-spliced gene and is not marked as duplicated despite appearing in multiple locations.

### 3.2. Chloroplast Genome Characteristics of Length and GC Content

A comparative analysis of chloroplast genome sizes was conducted across 28 Acanthaceae species. The total length of the complete chloroplast genomes was found to range from 144,133 bp to 153,783 bp. Among these, the longest genome was identified in *Sta. concinnula* (153,783 bp), followed by *Ch. longifolia* (152,594 bp), *Ap. knappiae* (152,457 bp), and *Ech. longzhouensis* (152,385 bp). The shortest genome was observed in *Str. cusia* (144,133 bp), with *Str. tonkinensis* (144,765 bp) and *Hyg. ringens* (146,373 bp) also exhibiting relatively short lengths (Table S1). Further comparison of the characteristic regional lengths within each chloroplast genome revealed the following ranges: the LSC region varied from 81,979 bp in *Run. pectinata* to 92,248 bp in *Str. tonkinensis*; the SSC region ranged between 16,626 bp (*Run. pectinata*) and 17,888 bp (*Ap. knappiae*); and the IR region exhibited variation from 17,328 bp (*Str. cusia*) to 25,646 bp (*Str. concinnula*). The comparative analysis indicated that the interspecific variation in chloroplast genome length was primarily attributable to expansions and contractions of the IR region, followed by the SSC region, whereas the LSC region remained relatively conserved. Analysis of guanine and cytosine (GC) content in the chloroplast genomes revealed a narrow range of variation across the 28 Acanthaceae species. The overall GC content ranged from 38.0% (observed in *D. montana*, *Hyp. forskaolii*, *Run. pectinata*, *L. incurva*, and *Sta. concinnula*) to 38.6% (*Ech. longzhouensis*), with a range of merely 0.6% (Table S2). However, distinct differences in GC content were observed among the characteristic regions. The IR region exhibited the highest GC content (43.1–46.1%), with *Str. cusia* showing the maximum value (46.1%) and *Run. pectinata* the minimum (43.1%). The LSC region followed with an intermediate GC content (36.0–36.9%), while the SSC region displayed the lowest values (31.8–33.2%). Notably, the IR region demonstrated the greatest variation in GC content (range: 3.0%), which was substantially higher than that of the SSC region (range: 1.4%) and the LSC region (range: 0.9%).

### 3.3. Repeat Sequences Analysis

**Figure 3:**
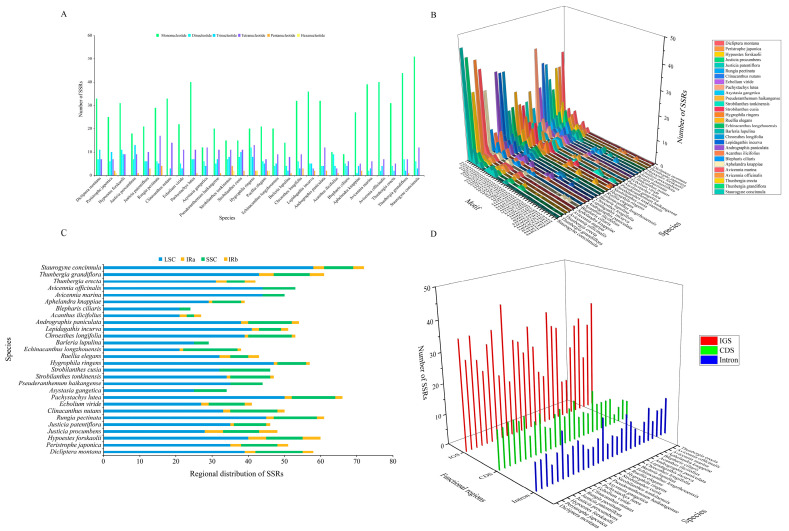
Identification of SSRs in the chloroplast genomes of 28 Acanthaceae species. (**A**) Detected nucleotide types. (**B**) Identified motif patterns, different colors represent distinct species within the Acanthaceae family. (**C**) Genomic distribution of SSR loci. (**D**) Functional classification of SSR-containing regions.

In this study, SSRs were identified across the chloroplast genomes of 28 medicinal species from the Acanthaceae family. A total of 24 to 72 SSR loci were detected per species (Figure 3). The composition was as follows: mononucleotide repeats (9–51, accounting for 37.50–70.83% of the total), dinucleotide repeats (0–12, 0–21.05%), trinucleotide repeats (2–13, 3.28–27.08%), tetranucleotide repeats (3–17, 5.88–27.87%), pentanucleotide repeats (0–4, 0–8.51%), and hexanucleotide repeats (0–2, 0–4.65%). Mononucleotide repeats were the most abundant, followed by tetranucleotide and trinucleotide repeats, while other repeat types accounted for relatively low proportions across all species. Notably, hexanucleotide repeats were only detected in *Pe. japonica*, *Str. tonkinensis*, *Hgy. ringens*, and *Rue. elegans*, and were absent in the remaining species (Figure 3A, Table S3). A total of 42 distinct SSR motifs were identified. All species exhibited a strong A/T bias in their repeat motifs, with the proportion of A/T motifs reaching up to 73.58%. The highest A/T content was observed in *Av*. *officinalis*, while the lowest was found in *Ac*. *ilicifolius*. The AT/AT and AAT/AAT motifs were also relatively common, accounting for 0–19.30% and 1.64–19.57% of the total SSRs, respectively. Other motifs, such as C/G, AG/CT, ATC/ATG, AAAC/GTTT, AACT/AGTT, AAGG/CCTT, and AAAAC/GTTTT, were present at lower frequencies and showed interspecific variation (Figure 3B, Table S4).

Analysis of SSR distribution across genomic regions revealed that 21–58 SSR loci were located in the LSC region (55.26–80.56%), 5–15 in the SSC region (11.63–39.47%), and 0–5 in the IR regions (0–10.42%). SSR loci were predominantly concentrated in the LSC region, followed by the SSC and IR regions. Specifically, *Sta. concinnula* possessed the highest number of SSR loci in the LSC region, followed by *Run. pectinata* and *T*. *grandiflora*, whereas *Ech. longzhouensis*, *Ac. ilicifolius*, and *Bl. ciliaris* had the fewest, each with 21 loci. *Ech. longzhouensis* contained the highest number of SSR loci in the SSC region, followed by *Str. cusia*, *D*. *montana*, and *Cl. nutans*. It is noteworthy that no SSRs were detected in the IR regions of *As. gangetica*, *Ps. haikangense*, *Str. cusia*, *Ba. lupulina*, *Bl. ciliaris*, *Av. marina*, and *Av. officinalis* (Figure 3C, Table S5). When categorized by functional regions, the IGS regions harbored the highest number of SSR loci (12–43, accounting for 44.44–65.15% of the total), significantly exceeding those in CDS regions (3–4, 7.32–29.17%) and intronic regions (3–15, 12.50–24.59%). *Pa. lutea* had the greatest number of SSRs in IGS regions, followed by *D*. *montana*, *Hyp. forskaolii*, *Cl. nutans*, and *Sta. concinnula*. In contrast, *Ba. lupulina*, *As. gangetica*, *Ech. longzhouensis*, and *T*. *erecta* possessed the fewest SSRs in IGS regions. These results indicate that SSRs are unevenly distributed within the chloroplast genomes of Acanthaceae species, with significant enrichment observed in the LSC and IGS regions (Figure 3D, Table S6).

### 3.4. Boundaries of Junction Sites Analysis

**Figure 4:**
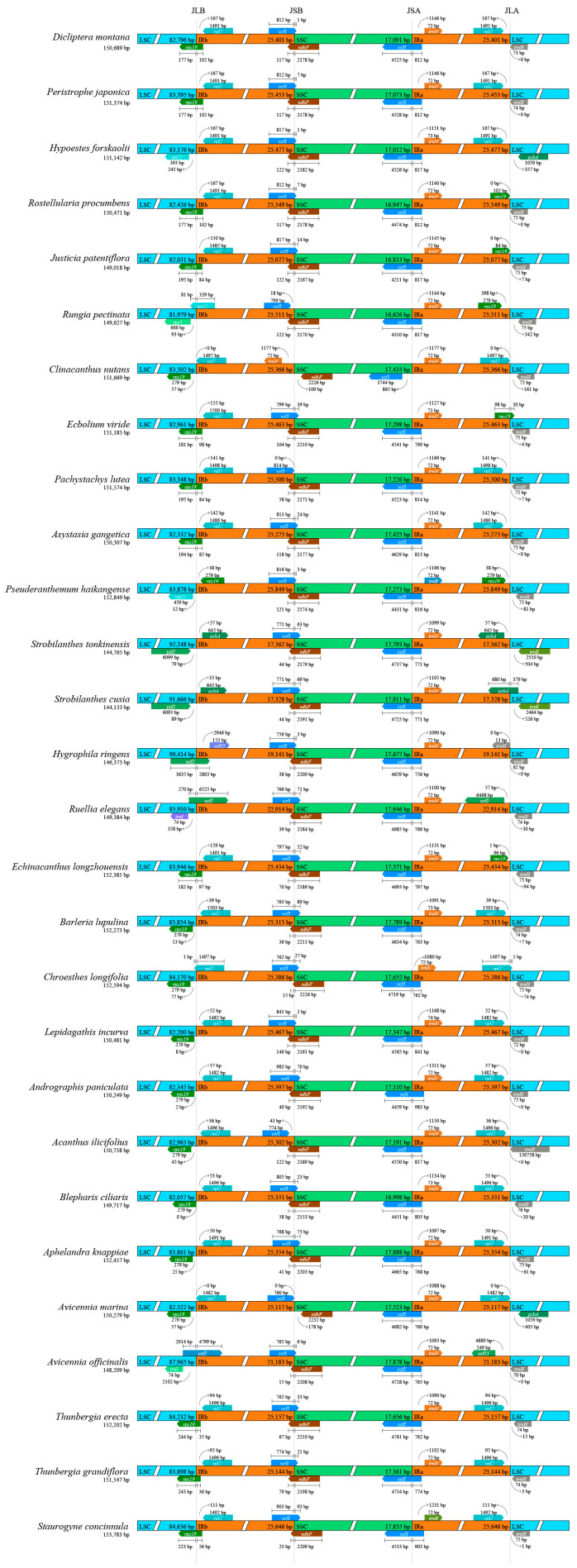
Comparative analysis of boundary regions in *T. grandiflora* and related species. In the comparative analysis of boundary regions, four distinct partitions are represented by different colors: blue indicates the length of the LSC region, orange-red denotes the two IR regions, and green corresponds to the SSC region. The junction sites between partitions are designated as JLB, JSB, JSA, and JLA, respectively. The arrows in the figure indicated the distances between the genes and their respective boundaries. Genes located at the boundary junctions are illustrated using pentagons of corresponding colors.

A comparative analysis of junction boundaries across the chloroplast genomes of 28 Acanthaceae species revealed distinct expansion at the junction sites of the four characteristic regions (LSC, IRb, SSC, and IRa). Notably, *Ps. haikangense* exhibited the most pronounced expansion, exceeding that of *T*. *grandiflora* by approximately 692 bp. Moderate expansions were also detected in *J*. *procumbens*, *Run. pectinata*, *Pa. lutea*, and *Sta. concinnula*, which were approximately 392 bp, 354 bp, 343 bp, and 489 bp longer, respectively, compared to *T. grandiflora*. In contrast, significant contraction of the IR region boundaries was identified in *Str. tonkinensis*, *Str. cusia*, *Hyg. ringens*, *Rue. elegans*, and *Av. officinalis*. Their IR regions were contracted by approximately 7795 bp, 7829 bp, 6015 bp, 2243 bp, and 3974 bp, respectively, relative to the remaining species with typical boundaries (Figure 4). A total of ten genes were involved in the boundary regions, including *rps19*, *rpl2*, *rpl22*, *rps3*, *psbA*, *ycf2*, *ycf15*, *trnI*, *trnN*, *ndhF*, *ycf1*, *trnR*, *trnH*, and *trnK*. At the LSC/IRb boundary, the junction site was located within the coding region of the *rps19* gene in *D*. *montana*, *Pe. japonica*, *J*. *procumbens*, *J. patentiflora*, *Ecb. viride*, *Pa. lutea*, *As. gangetica*, *Ech. longzhouensis*, *T*. *erecta*, *T. grandiflora*, and *Sta. concinnula*, positioned 177–244 bp from the LSC region and 35–102 bp from the IRb region. In *Cl. nutans*, *Ba. lupulina*, *Ch. longifolia*, *L*. *incurva*, *An. paniculata*, *Ac. ilicifolius*, *Ap. knappiae*, and *Av. marina*, the *rps19* gene was located to the left of the boundary, 2–77 bp from the LSC region. The *rpl2* gene was found on the right side of the boundary in 18 species, 36–167 bp from the IRb region. Notably, interspecific variation in gene composition was observed at this boundary. For instance, the *rps19* gene was absent in *Hyp. forskaolii*; both *rps19* and *rpl2* were absent in *Run. pectinata*, with *rps3* and *rpl22* appearing instead. In *Str. tonkinensis* and *Str. cusia*, *ycf2* and *psbA* were present; *ycf2* and *ycf15* were found in *Hyg. ringens*; and *ycf2* and *trnI* were identified in *Rue. elegans* and *Av. officinalis*. At the IRb/SSC boundary, in the analysis of results, the *ycf1* gene was annotated as a pseudogene in 27 species. Among these, the junction sites at this boundary were located within the coding region of the *ycf1* gene for 23 species. The length of the gene segment was 983–756 bp within the IRb region and 1–93 bp within the SSC region. The junction sites for *Run. pectinata*, *Pa. lutea*, *Ac. ilicifolius*, and *Av. marina* were positioned to the right of this boundary, at a distance of 0–18 bp from the SSC region. However, the *ycf1* gene was not annotated in *Cl. nutans* and is presumed to be lost. Furthermore, the junction sites at this boundary were situated within the coding region of the *ndhF* gene for 26 species. The segment of this gene spanned 15–146 bp in the IRb region and 2153–2226 bp in the SSC region. In contrast, the *ndhF* genes of *Cl. nutans* and *Av. marina* were located to the right of this boundary, at a distance of 109–178 bp from the IRb region. At the SSC/IRa boundary, the junction was located within the *ycf1* coding region in 26 species, with the gene spanning 4211–4761 bp in the SSC region and 762–983 bp in the IRa region. Unlike the *ycf1* at the IRb/SSC boundary, all *ycf1* genes here were functional. However, in *Ch. longifolia*, the *ycf1* gene was absent and was replaced by the *ndhF* gene. Furthermore, except for *Ps. haikangense* and *Sta. concinnula*, which had the *trnR* gene on the right side of this boundary, all other species possessed the *trnN* gene at this position, located 1080–1311 bp from the SSC region. At the IRa/LSC boundary, substantial variation in gene composition was observed. In 14 species, the boundary was flanked by *rpl2* and *trnH* genes, located 0–167 bp from the LSC region and 0–161 bp from the IRa region, respectively. In the 28 species, the boundary was flanked by *rps19* and *trnH*, positioned 0–398 bp from the LSC region and 0–342 bp from the IRa region. In *Hyp. forskaolii* and *Av. marina*, the flanking genes were *rpl2* and *psbA*; in *Str. tonkinensis* and *Str. cusia*, they were *psbA* and *trnK*; and in *Av. officinalis*, the junction site was formed by *ycf15* and *trnH*.

**Figure 5:**
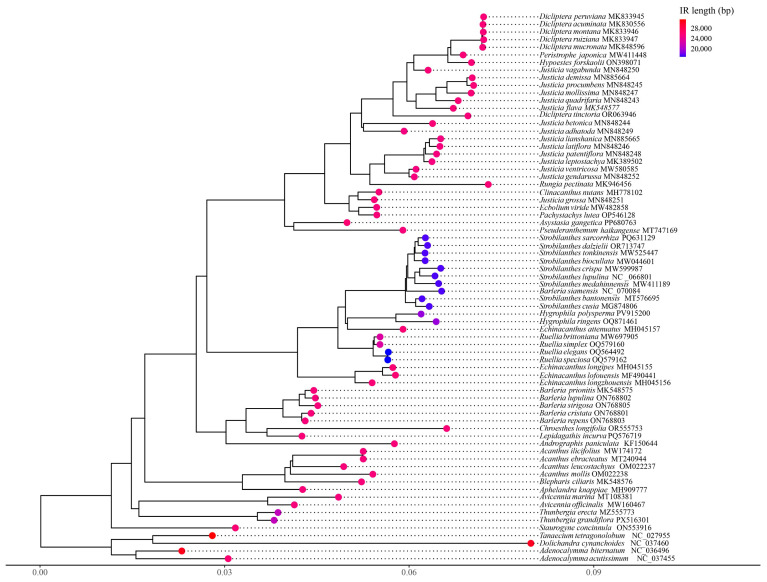
Mapping diagram of IR region length variation gradients onto the Phylogenetic tree of Acanthaceae. The legend in the upper right corner represents the IR region length, and the scale at the bottom represents patristic distances.

Mapping IR region lengths onto the phylogenetic tree revealed that IR length variation was not randomly distributed but tended to cluster within specific clades. Species exhibiting significant IR contraction grouped into one branch, while those with notable IR expansion formed a distinct evolutionary clade (Figure 5). A clear correspondence was observed between the contraction of IR length and the topological structure of the phylogenetic tree. In particular, species within the genus *Strobilanthes* exhibited significant contraction in their IR regions, which corresponded consistently with their tendency to cluster into an independent clade in the phylogenetic tree. This suggests that structural variation in the IR regions may be closely associated with the evolutionary divergence of this group. A Mantel test demonstrated a strong and statistically significant positive correlation between IR length differences and phylogenetic distances (Mantel statistic r = 0.368, *p* = 0.0001), indicating that more distantly related species showed greater divergence in IR length. These results suggest that IR length variation at least partially reflects evolutionary divergence among species. To further illustrate this relationship, a phylogenetic tree diagram was generated with a superimposed color gradient representing IR length variation, visually highlighting the correlation between IR length and phylogeny.

### 3.5. Whole Genome Comparative Analysis

**Figure 6:**
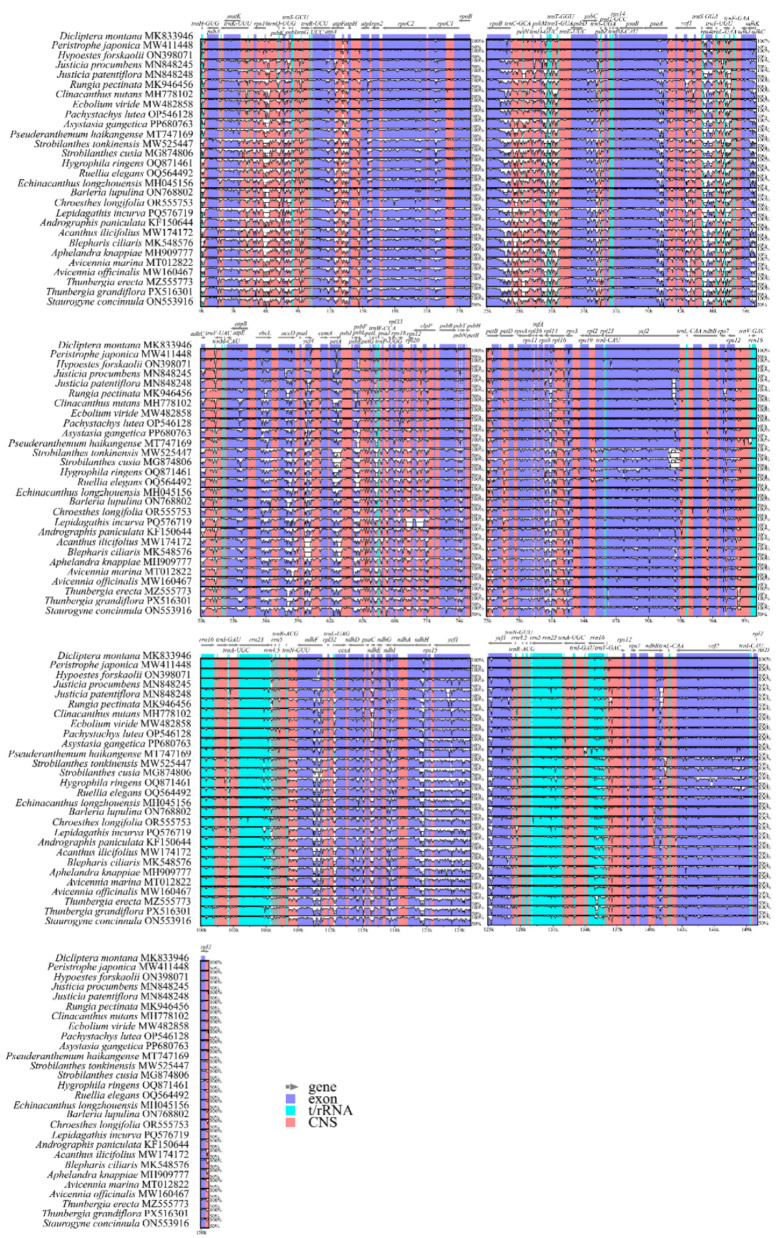
Global alignment analysis using mVISTA in 28 Acanthaceae species. The upper portion of the figure represents genes and their genomic locations, with arrows indicating transcriptional direction; dark blue denotes exons, light blue indicates tRNA/rRNA genes, pink represents non-coding regions, white serrations indicate regions of high variability, while smooth edges denote relatively conserved sequences.

Based on comparative chloroplast genome sequence analysis, variations were identified across Acanthaceae species (Figure 6). First, the degree of sequence variation was greater in non-coding regions than in protein-coding regions. Notably, elevated levels of variation were observed in several IGS regions, including *rps16*-*trnQ*-UUG, *psbK*-*psbI*, *atpF*-*atpH*, *rpoB*-*trnC*-GCA, and *trnS*-GGA-*rps4*, with all examined species exhibiting some degree of variation in these regions. Second, sequence variation was most pronounced in the LSC region, followed by the SSC region, while the IR regions displayed the lowest level of sequence divergence. This finding further supports the greater structural conservation of the IR regions during evolutionary processes.

### 3.6. Patterns of Synonymous Codon Usage

**Figure 7:**
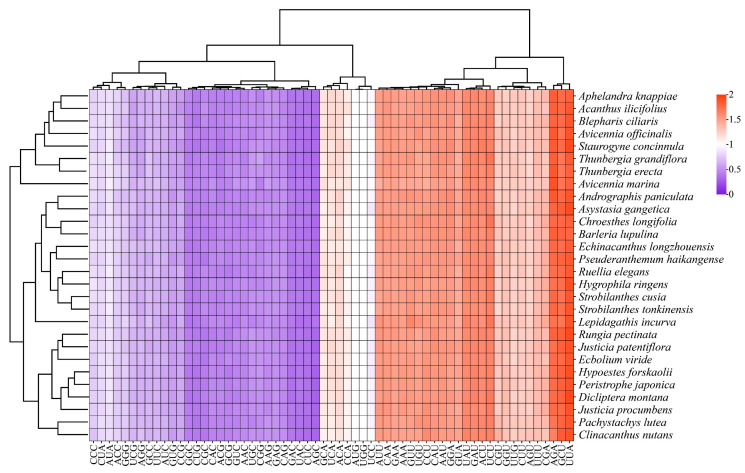
Analysis of synonymous codon usage in 28 Acanthaceae species. The RSCU values are indicated by squares of different colors.

In this study, RSCU analysis was conducted on the chloroplast genomes of 28 Acanthaceae species (Figure 7, Table S7). Since termination codons do not encode any amino acids, after the removal of stop codons UAA, UAG, and UGA. The most frequently used codon among the 28 species were AUU, which encodes isoleucine (Ile). The frequency of this codon ranged from 840 to 908, with a total of 24,449 uses across all species and an RSCU value ranging from 1.42 to 1.56. Conversely, the least frequently used codon was UGC, encoding cysteine (Cys), with a frequency ranging from 46 to 62 across different species, a total of 1553 uses across all species, and an RSCU value ranging from 0.43 to 0.55. Among the 61 codons encoding amino acids, 29 codons had an RSCU > 1, with RSCU values ranging from 1.02 to 1.94. Among them, 28 codons ended with A/U, and 1 ended with G, indicating that codons ending with A/U were the preferred codons in Acanthaceae species. Specifically, UUA, AGA, UCU, GCU, GAU, and UAU exhibited relatively high RSCU values (RSCU > 1.60), encoding leucine (Leu), arginine (Arg), serine (Ser), alanine (Ala), aspartic acid (Asp), and tyrosine (Tyr), respectively. Similarly, 30 codons had an RSCU < 1, with RSCU values ranging from 0.30 to 0.98. Among them, 28 codons ended with G/C, and two ended with A, indicating that codons ending with G/C were non-preferred codons in Acanthaceae species. Notably, the RSCU values of codons AUG and UGG were equal to 1, indicating that these two codons exhibited no codon bias.

The ENC results indicated that most chloroplast genes of Acanthaceae species deviated from the standard curve, being located below it, and the ENC values of all species were greater than 35 (Table S8). This suggested that codon usage bias in these species was generally weak, and these genes were classified as low-expression genes, implying that the codons might have been subjected to relatively weak selective pressures. An analysis of PR2 bias in the fourfold degenerate synonymous codons revealed that the coding genes were unevenly distributed across the four quadrants of the plot, with a higher density of genes in the lower-left quadrant (Table S9). This indicated a T/C bias at the third codon position in the chloroplast genomes of Acanthaceae species. Furthermore, the codon usage patterns in these chloroplast genomes were influenced not only by mutational bias but also by other factors, such as selective pressure. The neutral plot analysis further elucidated the mode of evolutionary forces. The GC3 content varied between 18.08% and 47.99%, while the GC12 content ranged from 32.61% to 57.55% (Table S10). The regression coefficients were low (R^2^ = 0.0024–0.0604), with *Av. marina* exhibiting the smallest R^2^ and *Hyp. forskaolii* the largest, indicating a weak correlation between GC12 and GC3. The distribution of gene points appeared scattered and showed no significant correlation with the diagonal, indicating substantial compositional differences in the third codon positions of the chloroplast genomes among Acanthaceae species. Furthermore, the slope of the regression curve ranged from 0.0514 to 0.2958, implying that Acanthaceae species were influenced to a lesser extent by mutational pressure but more significantly by natural selection.

### 3.7. Nucleotide Polymorphism Analysis

**Figure 8:**
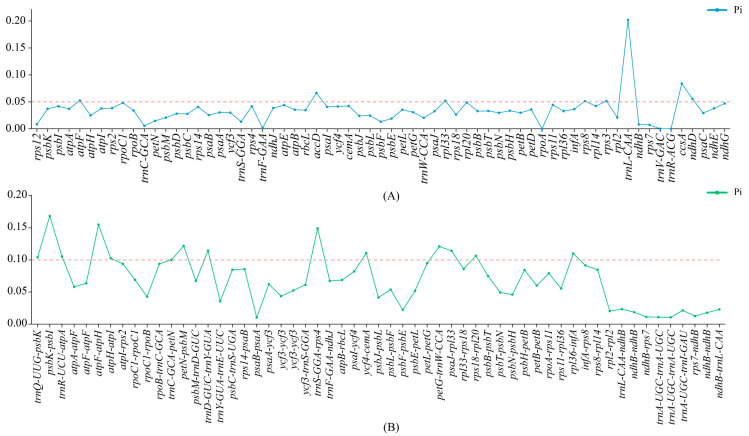
Nucleotide polymorphism analysis of genes and IGS regions in 68 Acanthaceae species. (**A**) Nucleotide diversity plot of genes (red dashed line: π > 0.05). (**B**) Nucleotide diversity plot of IGS (red dashed line: π > 0.10). In the figure, the red dashed lines represented the hypervariable regions identified through the screening process.

In this study, nucleotide diversity (π) analysis was performed on 65 shared genes and 57 shared intergenic spacers (IGS, including introns) from 68 complete chloroplast genomes of Acanthaceae species (Figure 8). Comparative analysis revealed that genetic variation was higher in the shared genes than in the IGS regions. Among the 65 genes, eight highly variable regions (π > 0.05) were identified (Figure 8A, Table S11): *trnL*-CAA (π = 0.20197), *ccsA* (π = 0.08401), *accD* (π = 0.06643), *ndhD* (π = 0.0556), *atpF* (π = 0.0525), *rpl33* (π = 0.05209), *rps3* (π = 0.05149), and *rps8* (π = 0.05142). In terms of genomic distribution, *accD*, *atpF*, *rpl33*, *rps3*, and *rps8* are located in the LSC region, whereas *trnL*-CAA, *ccsA*, and *ndhD* reside in the SSC region. Among the 57 shared IGS regions, 11 were identified as highly variable (π > 0.10) (Figure 8B, Table S12): *psbK*-*psbI* (π = 0.16842), *atpF*-*atpH* (π = 0.15499), *trnS*-GGA-*rps4* (π = 0.1492), *petN*-*psbM* (π = 0.12177), *petG*-*trnW*-CCA (π = 0.121), *trnD*-GUC-*trnY*-GUA (π = 0.11439), *psaJ-rpl33* (π = 0.11424), *ycf4*-*cemA* (π = 0.11064), *rpl36*-*infA* (π = 0.11), *rps18*-*rpl20* (π = 0.10644), and *trnR*-UCU-*atpA* (π = 0.10547). All of these highly variable IGS regions were located within the LSC region. The identified highly variable regions exhibited notable sequence polymorphism and may serve as ideal molecular markers for species identification within Acanthaceae.

### 3.8. Characterization of Selection Pressure on Coding Sequences

**Figure 9:**
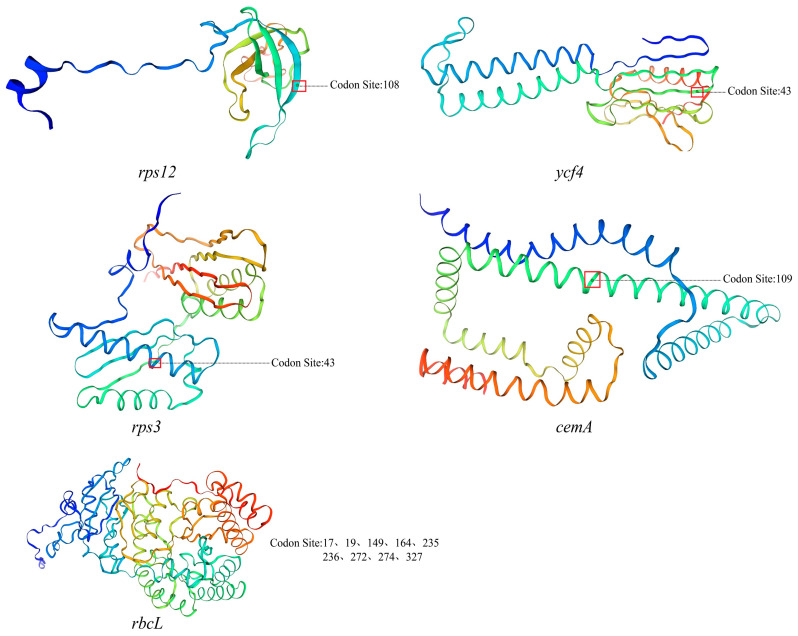
Predicting the spatial positions of positively selected sites within protein structures.

To investigate the adaptive evolutionary characteristics of the chloroplast genomes within Acanthaceae species, this study conducted Ka/Ks analysis on 62 conserved protein-coding genes across Acanthaceae species. The results revealed that most genes associated with photosynthesis (e.g., *atpA*, *atpB*, *atpE*, *atpF*, *psbB*, *psbC*, *psbD*, *ndhC*, *ndhK*, *ndhJ*) and self-replication (e.g., *rpl2*, *rpl20*, *rpl14*, *rpoB*, *rps3*, *rps7*, *rps12*) exhibited Ka/Ks values less than 1, indicating that these genes are under purifying selection. However, some genes showed Ka/Ks values greater than 0.6 (Table S13). To further investigate the evolutionary characteristics of the relevant genes, BUSTED and MEME analyses were subsequently employed. The BUSTED analysis revealed limited evidence of episodic diversifying selection among the Acanthaceae species across the 33 chloroplast coding sequences. Genome-wide statistically significant signals of positive selection (*p* < 0.05) were detected in five genes: *cemA*, *rbcL*, *rps3*, *rps12*, and *ycf4*. Although these genes exhibited complex evolutionary patterns, the vast majority of their sites remained under strong purifying selection. The MEME method was utilized to identify specific codon sites under positive selection. Episodic diversifying selection was detected at specific codon sites within all five of the aforementioned genes (Table S14). Among the positive selection sites mentioned above, codon site 108 of the *rps12* gene, condons 17, 149, 164, 235, 236, and 272 of the *rbcL* gene, and residue 43 of the *ycf4* gene were all identified as located within the coiled-coil domain. Residue 43 of the *rps3* gene, residue 274 of the *rbcL* gene, and residue 109 of the *cemA* gene were detected within the α-helix domain. Meanwhile, residues 19 and 327 of the *rbcL* gene were found to be situated in the strand domain (Figure 9).

### 3.9. Reconstructed Phylogenetic Relationships

**Figure 10:**
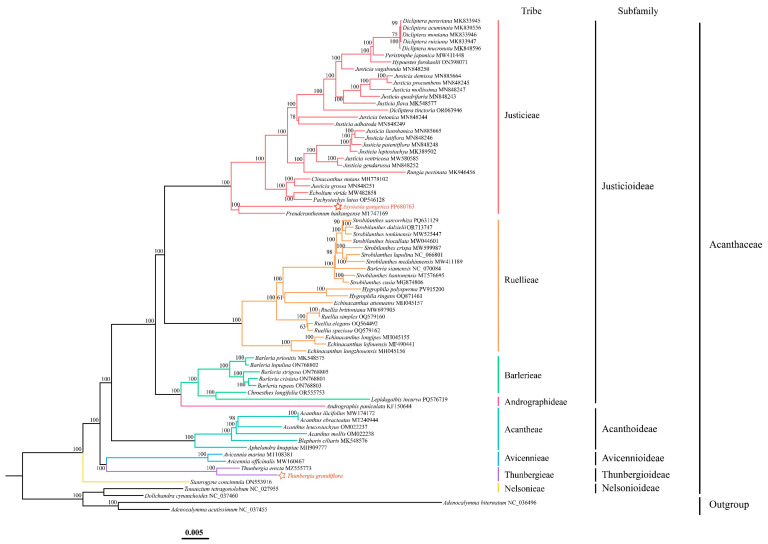
Phylogenetic tree of Acanthaceae. The values on the branches in the figure represent maximum likelihood bootstrap support values (BS), and branches are color-coded to represent different tribes. Species marked with an orange-red asterisk represent those sequenced and assembled in this study, while species marked with a red asterisk indicate those previously sequenced by our research group and already deposited in the NCBI database.

To elucidate the phylogenetic relationships within the Acanthaceae family, this study constructed a ML tree based on complete chloroplast genome sequences from 68 Acanthaceae species encompassing 24 genera. Additionally, an ML tree was generated using 52 shared CDS (Figure S1). The results indicated that the topologies of the two trees were highly consistent; therefore, only the tree based on complete chloroplast genomes is presented in the main text (Figure 10). At the subfamily level, the phylogenetic analysis revealed that Acanthaceae comprises five major subfamilies: Justicioideae, Acanthoideae, Avicennioideae, Thunbergioideae, and Nelsonioideae. Justicioideae and Acanthoideae were resolved as sister groups with strong bootstrap support (BS = 100). Similarly, Avicennioideae and Thunbergioideae formed a highly supported sister clade (BS = 100). Nelsonioideae was positioned at the base of the Acanthaceae phylogeny, representing the earliest-diverging lineage and serving as the sister group to all other subfamilies. At the tribal level, the phylogenetic tree encompassed eight tribes. Within Justicioideae, the tribes Justicieae and Ruellieae formed a major clade as sister groups, which clustered together with Barlerieae and Andrographideae. The tribes Avicennieae and Thunbergieae were recovered as strongly supported sister groups, belonging to Avicennioideae and Thunbergioideae, respectively. Nelsonioideae represents the earliest-diverging lineage of Acanthaceae, while Thunbergieae and Avicennieae form a well-supported clade that is sister to the core Acanthaceae (sensu stricto). At the genus and species levels, within the Thunbergia, *T. grandiflora* and *T. erecta* were resolved as sister species. These species formed a larger clade with members of the *Avicennia*. Furthermore, *As. gangetica*, sequenced in a previous study by our research group, was placed within the tribe Justicieae. This species exhibited a relatively late divergence and was phylogenetically distant from the tribe Thunbergieae.

### 3.10. Estimated Divergence Time Framework

Convergence of the analysis was assessed using Tracer v1.7 [94], and all parameters showed ESS greater than 200, indicating satisfactory convergence. The divergence time estimation revealed that the stem group of Acanthaceae originated in the late Paleocene, approximately 66 million years ago (Ma), with a 95% credibility interval (CI) of 50.02–85.87 Ma (Figure S2). Subsequent diversification occurred during the middle to late Eocene, followed by a rapid radiation. Specifically, the divergence between *T. grandiflora* and *T. erecta* was estimated at approximately 11 Ma. The most recent common ancestor of Thunbergieae and Avicennieae was dated to approximately 34 Ma (95% CI: 29.51–37.55 Ma), the crown group of Acanthaceae was dated to approximately 46 Ma (95% CI: 38.31–55.92 Ma). Based on the molecular clock analysis, the hooked retinacula—an evolutionary key innovation in core Acanthaceae—likely originated during the Eocene at approximately 37.56 Ma.

## 4. Discussion

Acanthaceae is primarily distributed in tropical to subtropical regions and possesses abundant resources of traditional Chinese medicine (TCM). Notable examples include the widely recognized *Str*. *cusia*, whose dried stems and roots are used medicinally as “Nan Ban Lan Gen” (Southern Strobilanthes Root) [95], and *An*. *paniculata*, renowned as the “herbal antibiotic” in TCM due to its richness in andrographolide—a diterpenoid compound with significant antibacterial activity [96]. Other notable medicinal species within the family include *Pe*. *japonica*, *J*. *procumbens*, and *As*. *gangetica* [47,51]. Despite this diversity, many medicinal resources in Acanthaceae remain systematically unidentified and underutilized. To address this, a comparative genomic analysis was conducted on 28 Acanthaceae species with documented use in classical pharmacopeias to investigate the genomic similarity and degree of variation among these medicinal plants. This work provides a new genomic perspective for the exploration and investigation of medicinal plants in Acanthaceae.

To elucidate the characteristics of chloroplast genomes in Acanthaceae within a closer phylogenetic context, this study selected Lamiaceae Martinov, Plantaginaceae Juss., Scrophulariaceae Juss., and Linderniaceae Borsch, K. Müll. & Eb. Fisch. within the order Lamiales as comparative subjects, based on the availability of relatively comprehensive data. Comparative analysis reveals that the chloroplast genomes of Acanthaceae exhibit a unique evolutionary pattern characterized by “dynamic macrostructure yet deeply conserved micro-elements.” Acanthaceae demonstrates significantly higher evolutionary plasticity in genome architecture. The wide variation in chloroplast genome size primarily stems from frequent and pronounced boundary contraction and expansion events in the IR regions. For instance, severe contractions of up to approximately 8 kb were detected in *Strobilanthes* [54,95], *Hygrophila*, and the mangrove species *Av. marina* [97], resulting in the transition of key genes such as *rps19* and *ycf1* from dual-copy to single-copy status, which may profoundly affect their gene dosage effects and expression regulation. This stands in sharp contrast to the generally conservative patterns observed in the related families. The IR regions in Lamiaceae exhibit remarkable structural stability with typically narrow genomic size variation [98,99,100,101,102]; studies on Plantaginaceae also indicate relatively fixed IR boundaries [103,104], Linderniaceae also displays a conserved IR structure [105,106]. However, in species of the genus *Plantago* L., the expansion of the IR regions has resulted in the occurrence of large-scale inversion events [91]. This discrepancy underscores the unique strategy of Acanthaceae in driving genomic differentiation through active IR recombination. However, a deeply conserved pattern is shared among these families in terms of base composition. The overall GC content of Acanthaceae is similar to that of representative Lamiaceae species and some Orobanchaceae Vent. groups, and the characteristic high GC content in the IR regions is prevalent among core Lamiales lineages [107,108], suggesting widespread selective pressure to maintain the structural stability of this region. In contrast to the variability at the macrostructural level, profound phylogenetic conservation is observed at the level of genes and introns. The composition of the core genome is highly consistent, with all encoding approximately 132 genes. Notably, the distinctive double-intron structure of key genes (e.g., *ycf3* and *clpP*) is completely conserved in Acanthaceae, Lamiaceae, and Linderniaceae. This feature is highly likely a synapomorphy for Lamiales [109,110], strongly implying that this complex structure is crucial for the proper function of these genes.

In terms of SSR characteristics, Lamiales lineages also show both convergent and specific evolutionary trends. The total number of SSR loci in Acanthaceae is similar to that found in some Lamiaceae and Scrophulariaceae species. In composition, a strong A/T bias is a common trend; for instance, species from closely related families are dominated by A/T-type repeat units, reflecting similar mutational pressures or selective environments [111,112]. Concurrently, the distribution pattern of SSR enrichment in non-coding regions is highly consistent across these families, aligning with their general evolutionary pattern [113]. In conclusion, through comparison with closely related families within Lamiales, this study clarifies that while maintaining high conservation in its core genetic machinery, the chloroplast genomes of Acanthaceae have undergone more active structural variation in their IR regions. This pattern of “conservation within dynamics” may constitute an important genomic foundation for the rich species diversity and ecological adaptability of Acanthaceae. Future research should integrate more functional genomic evidence to further elucidate the specific adaptive significance and evolutionary drivers of the structural dynamics of the IR regions.

Variation in chloroplast genome size has been recognized as a common evolutionary phenomenon, primarily attributed to the contraction and expansion of IR regions [114,115]. Although chloroplast genome boundaries are generally conserved in plants, some species exhibit inversions in boundary regions, along with frequent gene loss and expansion/contraction events in angiosperms [116]. This study identified extreme and asymmetric structural variations in the IR regions of chloroplast genomes across Acanthaceae species. On one hand, moderate expansions of several hundred base pairs were observed in species such as *Ps. haikangense*. On the other hand, severe contractions of up to approximately 8 kb were detected in lineages including *Strobilanthes*, *Hygrophila*, and the mangrove species *Av. officinalis*. These contractions likely resulted from repeat mediated non homologous recombination mechanisms, leading to the transition of key genes (e.g., *rps19*, *rpl2*, *rpl23*, *ycf2*, *ycf1*) from dual copy to single copy status, which may profoundly alter their gene dosage effects and expression regulation [54,95], this phenomenon has also been detected in plants of the family Araceae [117]. Notably, IR contractions were not randomly distributed phylogenetically but were concentrated in specific clades, suggesting that they may represent synapomorphies for these groups. The pronounced contraction observed in *Strobilanthes* aligns with previously reported genome wide compaction trends in this genus. Additionally, the contraction detected in *Avicennia* species may reflect part of a chloroplast genome optimization strategy in mangrove plants to adapt to high salinity and low oxygen coastal environments, potentially by streamlining the genome to reduce replicative burden and enhance adaptive capacity [98]. Compared with the well documented phenomenon of complete IR loss in Fabaceae [118,119], the IR contractions revealed in this study represent another significant mode of structural dynamics in chloroplast genome evolution. However, the precise physiological and adaptive implications of these structural variations await further validation through integrated transcriptomic and population genomic analyses.

The mVISTA global alignment analysis effectively visualized sequence similarities and divergences among different species, while nucleotide polymorphism analysis identified genes or regions with high variability [120]. Through mVISTA and DnaSP analyses, it was found that non-coding regions in Acanthaceae species exhibited greater sequence variation than coding regions, which was consistent with the predominant distribution of SSR loci in non-coding regions. Although DNA barcoding techniques utilizing sequences such as *ITS*, *matK*, and *rbcL* have been widely applied for species identification [121,122], they still face challenges in distinguishing morphologically similar species. In contrast, chloroplast genomes, characterized by slower evolutionary rates compared to nuclear genes, have proven valuable for phylogenetic reconstruction and species discrimination. This study identified several highly variable regions, such as *accD*, *atpF*, *rpl33*, *psbK-psbI*, *atpF-atpH*, and *trnS-GGA-rps4*, that contain numerous polymorphic sites, indicating their potential as effective DNA barcodes for the identification of Acanthaceae species.

Codon usage bias, reflecting non-random utilization of synonymous codons, represents a widespread phenomenon in the plant kingdom. This bias plays a crucial role in determining gene expression levels and cellular functions [123,124]. Investigation of codon usage patterns contributes significantly to understanding genetic architecture and evolutionary processes in organisms. This study revealed a significantly higher abundance of A and T bases compared to G and C at the third codon position, demonstrating a distinct A/T bias. Furthermore, among the 28 codons with RSCU > 1, all terminated with A/T, further emphasizing this positional preference at the third codon site. These findings align with previous reports in *Bauhinia* L., *Gynostemma* Blume, and *Astragalus* L. [125,126,127]. Multiple factors influence codon usage bias, including gene expression levels, gene length, tRNA abundance, and mutational tendencies [128]. While all these elements contribute to codon preference, the analyses highlighted the predominant roles of mutational pressure and natural selection. ENC-plot and PR2-plot evaluations demonstrated that mutational pressure exerted minimal influence on codon usage preference in Acanthaceae species, whereas natural selection served as the dominant factor. These results are consistent with findings in chloroplast coding sequences of *Epimedium* L., *Panicum* L., and *Arachis* L. [129,130,131]. Neutrality analysis further confirmed natural selection as the primary determinant shaping codon usage bias in Acanthaceae species.

Selection pressure in molecular pharmacognosy is primarily reflected in the study of evolutionary patterns of positively selected genes, uncovering the genetic basis of secondary metabolic pathways in medicinal plants, the molecular mechanisms underlying variations in pharmacologically active compounds, and the impact of environmental adaptation on the quality of medicinal materials [132,133]. Positive selection has been recognized as playing a crucial role in organismal adaptation to diverse environments. To investigate the characteristics of adaptive evolution at the sequence level in the chloroplast genomes of Acanthaceae, positive selection analyses were conducted. The results revealed that signals of positive selection were not widespread across the entire genome but were specifically concentrated at particular amino acid sites within a limited set of critical genes [134]. The genes identified under positive selection include: *rbcL* (involved in carbon assimilation within the photosynthetic pathway) [135], *rps3* and *rps12* (encoding ribosomal subunit proteins essential for chloroplast self-replication) [136], *cemA* (potentially involved in transmembrane ion transport and associated membrane functions) [136], and *ycf4* (whose function is not yet fully characterized but is known to be essential for Photosystem I assembly) [137]. Together, these genes form a functional network closely linked to the core physiological processes of “energy capture and organelle homeostasis maintenance.” Notably, the positively selected sites identified in genes such as *rbcL* using the MEME method were predominantly located near known protein functional domains or molecular interaction interfaces. For instance, several selected sites in the *rbcL* gene are situated adjacent to its substrate-binding pocket, suggesting that adaptive evolution may fine-tune the CO_2_ affinity or catalytic efficiency of Rubisco [138], thereby optimizing carbon fixation under varying light and temperature conditions [139]. This evolutionary pattern—characterized by “localized, episodic positive selection nested within a background of strong purifying selection”—appropriately illustrates how chloroplast genomes, while strictly conserving essential life functions, can still acquire adaptive advantages through subtle modifications of key proteins [140].

The maximum likelihood trees constructed based on complete chloroplast genomes and CDS in this study exhibited highly congruent topologies. Both trees consistently supported the division of Acanthaceae into five major subfamilies (Justicioideae, Acanthoideae, Avicennioideae, Thunbergioideae, and Nelsonioideae), encompassing eight tribes [32]. Compared to previous studies, this research systematically incorporated key genera such as *Hypoestes*, *Ecbolium*, *Pachystachys*, *Asystasia*, *Hygrophila*, *Chroesthes*, and *Lepidagathis* into the phylogenetic framework for the first time, significantly expanding the taxonomic coverage of phylogenetic studies in Acanthaceae. This expansion not only strengthened the statistical support for key nodes but also provided a more robust data foundation for elucidating evolutionary relationships among subfamilies and tribes. In the specific phylogenetic relationships, Justicioideae and Acanthoideae formed a stable sister group, while Avicennioideae and Thunbergioideae constituted another highly supported clade. Nelsonioideae was positioned at the base of Acanthaceae, supporting its status as the earliest diverging subfamily. At the tribal level, the sister relationship between Justicieae and Ruellieae was confirmed, and the newly included genus *Asystasia* was consistently placed within Justicieae, validating its traditional taxonomic position. The phylogenetic placements of genera such as *Hygrophila* and *Chroesthes* provided critical insights for further investigating their morphological evolution and ecological adaptations. However, some intergeneric relationships within certain tribes still exhibited low support or conflicts, suggesting that Acanthaceae may have undergone rapid radiative evolution or reticulate evolutionary events in its evolutionary history [141]. These complexities indicate that relying solely on chloroplast genome data may be insufficient to fully resolve the deep phylogenetic relationships within this group. Although chloroplast genomes are widely used in plant phylogenetic reconstruction due to their conserved sequences and high information content [142,143], their application to complex groups like Acanthaceae presents inherent limitations that must be carefully considered when interpreting results: in most angiosperms, chloroplast genomes are maternally inherited, recording only maternal lineage history [144]. This may result in chloroplast-based phylogenetic trees failing to reflect the complete evolutionary history of species, particularly in groups with paternal gene flow [145], asymmetric hybridization, or sex-biased dispersal [146]. Thus, chloroplast trees may represent “maternal gene trees” rather than “species trees” that reflect true species relationships [147]; multiple hybridization events are known within Acanthaceae [148]. Hybridization can lead to the “capture” of chloroplast genomes by one parent, resulting in chloroplast introgression across generations [149]. Such cross-species genome flow may cause chloroplast phylogenetic relationships to deviate from actual species divergence history, leading to “anomalous” relationships in the phylogenetic tree (e.g., distantly related species clustering together). The low support or conflicts with morphological classifications observed at certain nodes in this study may indicate undetected historical hybridization or introgression events; during rapid radiative divergence, ancestral genetic polymorphisms may be passed to different descendant lineages without sufficient time for complete sorting, leading to incongruence between gene trees and species trees—a phenomenon known as incomplete lineage sorting (ILS) [150]. Multiple subfamilies and tribes in Acanthaceae may have undergone rapid diversification over short periods [151], making ILS a potential cause of unresolved or conflicting phylogenetic relationships [152]; this study also identified significant structural variations (expansions/contractions) in the IR regions of Acanthaceae chloroplast genomes [95]. Such large-scale structural changes may affect sequence alignment accuracy and introduce noise into sequence similarity-based phylogenetic reconstruction due to their independent evolutionary histories.

Based on publicly available complete chloroplast genome data of Acanthaceae, this study established the most comprehensive phylogenetic framework of Acanthaceae chloroplast genomes to date, laying an important foundation for understanding subfamilial and tribal relationships within the family. However, the genetic characteristics of chloroplast genomes imply that their phylogenetic signals may be biased [153]. To further clarify the complex evolutionary history of Acanthaceae, future studies should: (1) integrate nuclear gene sequences or reduced-representation genomic data to reconstruct species trees, overcoming the limitations of uniparental inheritance and ILS; (2) employ phylogenetic network analysis methods to explicitly detect and quantify the history and intensity of hybridization and introgression events [154]; (3) conduct population genomic sampling at intra- and interspecific levels to trace gene flow history and distinguish the effects of natural selection and genetic drift on chloroplast genome distribution patterns; and (4) perform multidimensional correlation analyses combining key morphological traits, biogeographic distributions, and ecological adaptation data to explore the coevolutionary mechanisms between evolutionary history and phenotypic diversification. Through the integration of multiple lines of evidence and cross-validation of analytical methods, a deeper and more systematic understanding of the phylogenetic patterns, evolutionary dynamics, and mechanisms of diversity formation in Acanthaceae can be achieved. The stem group of Acanthaceae was inferred to have originated in the late Paleocene, approximately 66 Ma. The warm and humid global climate conditions prevailing from the Paleocene to the Eocene may have provided a suitable environmental context for the establishment of early lineages of Acanthaceae and their initial diversification during the middle to late Eocene. The results indicate that the crown group of Acanthaceaeoriginated during the Eocene (approximately 46 Ma). Taxa from this period uniformly lacked a hooked retinacula structure. In contrast, deep intertribal divergence events, such as the split between Thunbergieae and Avicennieae around 34 Ma, extended into the Oligocene. From this period onward, retinacula gradually appeared in the morphology of Acanthaceae species. The Eocene-Oligocene Transition (EOT, ~34 Ma), a key geological period characterized by significant global cooling and the formation of the Antarctic ice sheet, broadly coincides temporally with events such as the closure of the Sea of Tethys [155] and the uplift of the Qinghai–Tibet Plateau [156]. These geological processes profoundly reshaped regional topography and climate patterns, potentially intensifying the Asian monsoon system and expanding tropical arid habitats. The resulting increase in environmental heterogeneity likely drove the differentiation of various Acanthaceae lineages as they adapted to distinct ecological niches. Furthermore, the divergence of species within the genus *Thunbergia* (between *T. grandiflora* and *T. erecta*) as well as most living species in Acanthaceae was estimated at approximately 11 Ma, during the Miocene. The Miocene was a period of active tectonism and pronounced climatic change, marked particularly by an enhanced Asian monsoon and increased seasonal aridity [157]. These changes may have caused habitat fragmentation [158], thereby promoting speciation in isolated populations of climbing herbs or shrubs such as *Thunbergia* following the formation of (sub)tropical forests. Notably, the Paleocene origin of inferred in this study is earlier than estimates from some studies based on limited gene fragments, which often suggested an Eocene origin [91,159,160]. This discrepancy may stem from the use of complete chloroplast genome data in the present study, which provides a greater number of informative sites and improves estimation accuracy. Additionally, the integration of four Acanthaceae fossil calibration points in this study further enhanced the reliability of the dating results. Consequently, the divergence time framework established here provides a more robust temporal calibration for further investigating the evolutionary history of Acanthaceae and indeed the entire order Lamiales during the Cenozoic.

These phylogenetic findings and divergence time estimates carry significant implications for biodiversity conservation. The identification of distinct evolutionary lineages within *Thunbergia*, coupled with the recognition of their unique evolutionary histories spanning millions of years, underscores the importance of conserving not just individual species but the full spectrum of phylogenetic diversity within the genus. The historical habitat fragmentation during the Miocene that drove speciation in *Thunbergia* finds a modern parallel in the ongoing anthropogenic habitat fragmentation threatening these species today [161]. The study provides crucial genetic resources for assessing population genetic diversity and delineating evolutionary significant units, which are fundamental for developing targeted conservation strategies. Furthermore, the chloroplast genomic markers identified here offer practical tools for monitoring genetic erosion and prioritizing conservation efforts for *Thunbergia* species, particularly those with medicinal value that face increasing harvesting pressures. Integrating this evolutionary perspective with conservation planning ensures the preservation of both the taxonomic and genetic diversity that has evolved over millennia in this ecologically and medicinally important group.

## 5. Conclusions

This study provides a comprehensive analysis of chloroplast genome evolution in Acanthaceae by integrating phylogenetic reconstruction, structural comparison, and selection pressure assessment. The phylogenetic tree constructed from 68 species offers robust support for the classification of Acanthaceae into five major subfamilies (Justicioideae, Acanthoideae, Avicennioideae, Thunbergioideae, and Nelsonioideae) and eight tribes, clarifying key relationships within the family. Furthermore, significant structural variations were detected in the IR regions of chloroplast genomes of specific lineages such as *Strobilanthes* and the mangrove-adapted *Avicennia*, suggesting that chloroplast genome architecture may be influenced by lineage-specific evolutionary pressures and potentially linked to ecological adaptation. Additionally, positive selection signals were identified in several key functional genes, including *rbcL*, *rps3*, *rps12*, *cemA*, and *ycf4*, revealing molecular mechanisms underlying adaptive evolution in Acanthaceae. These genes have undergone fine-tuned adaptive modifications at critical sites while maintaining overall functional conservation. The prevalent dominance of mononucleotide A/T repeats in SSR analysis further highlights distinctive genomic features of this family. In summary, this study advances the understanding of the dynamic evolution of chloroplast genomes in Acanthaceae, demonstrating how structural variations, repetitive sequences, and coding sequence evolution collectively drive species diversification within the family. This work also establishes a genomic foundation for future species identification, prioritization of endangered species conservation, and sustainable utilization of medicinal resources in Acanthaceae, providing scientific support for the protection of threatened species in rapidly changing tropical habitats.

## Figures and Tables

**Table 1:** Functional annotation of chloroplast genes in *Thunbergia grandiflora*.

Category for Genes	Group of Genes	Name of Genes	Total
Self-replication	Large subunit of ribosome	*rpl14*, *rpl16* *, *rpl2* *^, a^, *rpl20*, *rpl23* ^a^, *rpl32*, *rpl33*, *rpl36*	10
	Small subunit of ribosome	*rps11*, *rps12* *, *rps14*, *rps15*, *rps16* *, *rps18*, *rps19*, *rps2*, *rps3*, *rps4*, *rps7* ^a^, *rps8*	13
	DNA dependent RNA polymerase	*rpoA*, *rpoB*, *rpoC1* *, *rpoC2*	4
	rRNA genes	*rrn16* ^a^, *rrn23* ^a^, *rrn4.5* ^a^, *rrn5* ^a^	8
	tRNA genes	*trnA-UGC* *^,a^, *trnC-GCA*, *trnD-GUC*, *trnE-UUC*, *trnF-GAA*, *trnG-GCC*, *trnG-UCC* *, *trnH-GUG*, *trnI-CAU* ^a^, *trnI-GAU* *^,a^, *trnK-UUU* *, *trnL-CAA* ^a^, *trnL-UAA* *, *trnL-UAG*, *trnM-CAU*, *trnN-GUU* ^a^, *trnP-UGG*, *trnQ-UUG*, *trnR-ACG* ^a^, *trnR-UCU*, *trnS-GCU*, *trnS-GGA*, *trnS-UGA*, *trnT-GGU*, *trnT-UGU*, *trnV-GAC* ^a^, *trnV-UAC* *, *trnW-CCA*, *trnY-GUA*, *trnfM-CAU*	37
Photosynthesis	Photosystem I	*psaA*, *psaB*, *psaC*, *psaI*, *psaJ*	5
	Photosystem II	*psbA*, *psbB*, *psbC*, *psbD*, *psbE*, *psbF*, *psbH*, *psbI*, *psbJ*, *psbK*, *psbL*, *psbM*, *psbN (pbf1)*, *psbT*, *psbZ (lhbA)*	15
	NADPH dehydrogenase	*ndhA* *, *ndhB* *^, a^, *ndhC*, *ndhD*, *ndhE*, *ndhF*, *ndhG*, *ndhH*, *ndhI*, *ndhJ*, *ndhK*	12
	Cytochrome b/f complex	*petA*, *petB* *, *petD* *, *petG*, *petL*, *petN*	6
	Subunits of ATP synthase	*atpA*, *atpB*, *atpE*, *atpF* *, *atpH*, *atpI*	6
	Large subunit of Rubisco	*rbcL*	1
	Photosynthesis assembly genes	*ycf3 (pafI)* *, *ycf4 (pafII)*	2
Other genes	Protease	*clpP* *	1
	Maturase	*matK*	1
	Envelop membrane protein	*cemA*	1
	Subunit of Acetyl-CoA-carboxylase	*accD*	1
	C-type cytochrome synthesis gene	*ccsA*	1
	Translation initiation factor	*infA*	1
	Conserved open reading frames	*ycf2* ^a^	2
	Pseudogenes	*rpl22* Ψ, *ycf15* Ψ ^a^, *ycf1* Ψ ^a^	5
		Total number of genes	132

Note: *, ^a^, and Ψ indicate genes containing introns, duplicated genes in inverted repeat (IR) regions, and pseudogenes, respectively. The *rps12* gene is a trans-spliced gene and is not marked as duplicated despite appearing in multiple locations.

## Data Availability

The chloroplast genome sequence supporting this study has been deposited into GenBank (National Center for Biotechnology Information) with the accession number PX516301.1. The associated BioProject ID is PRJNA1337428, and the BioSample identifier is SAMN52217823. The corresponding SRA number is SRR35730976.

## Supplementary Materials

The following supporting information can be downloaded at: https://www.mdpi.com/article/10.3390/biology15020137/s1, Table S1: Lengths of the four characteristic regions; Table S2: GC Content; Table S3: SSR loci; Table S4: 42 distinct motif types; Table S5: SSR distribution regions; Table S6: SSR distribution functional regions; Table S7: Analysis of synonymous codon usage; Table S8: The effective number of codons analysis; Table S9: The parity rule 2 plot analysis; Table S10: The Pi value of genes; Table S11: The Pi value of intergenic spacer regions; Table S12: The effective number of codons analysis; Table S13: Analysis of selection pressure; Table S14: Analysis of Positive selection; Figure S1: Phylogenetic tree constructed based on CDS; Figure S2: Phylogenetic tree of Acanthaceae.
