# Supplementary material for: Comparative Chloroplast Genomics of Acanthaceae with a Focus on Medicinal Plant Thunbergia grandiflora Roxb.: Unveiling Adaptive Evolution, Diversification Mechanisms and Phylogenetic Relationships
Source: Biology (Basel). 2026 Jan 13;15(2):137. doi: 10.3390/biology15020137 (PMC12837636; doi:10.3390/biology15020137)
Supplement: Supplementary file 1 [file biology-15-00137-s001.zip › Figure S2.pdf]

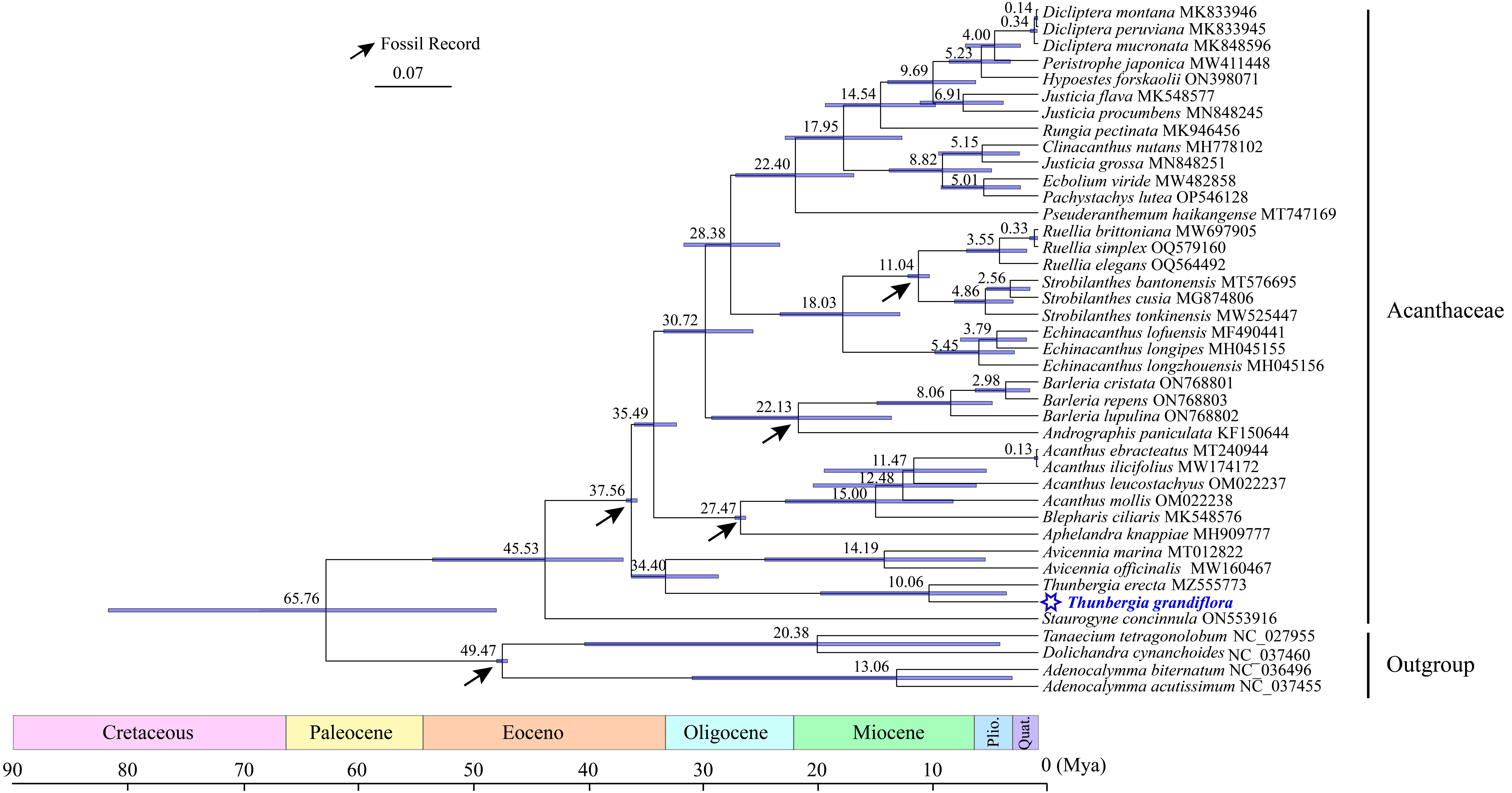

Figure S2. Estimation of divergence times in the Acanthaceae. The blue outline denotes the 95% confidence interval; the bottom axis represents the divergence time scale.
